# Innovative use of a stent retriever for temporary revascularization in acute internal carotid artery dissection

**DOI:** 10.1016/j.radcr.2024.06.077

**Published:** 2024-07-26

**Authors:** Masahiro Sugihara, Atsushi Fujita, Takeshi Kondoh, Yoshiyuki Takaishi, Hirotomo Tanaka, Takashi Sasayama

**Affiliations:** a Department of Neurosurgery, Shinsuma General Hospital, 3-1-14 Kinugake-cho, Suma-ku, Kobe 654-0048, Japan; b Department of Neurosurgery, Kobe University Graduate School of Medicine, 7-5-1 Kusunoki-cho, Chuo-ku, Kobe 650-0017, Japan

**Keywords:** Angioplasty, Thrombectomy, Ischemic stroke, Revascularization, Arterial dissection

## Abstract

Internal carotid artery dissection is rare but can be a cause of stroke in young people. In a case of revascularization for stroke associated with internal carotid artery dissection, we initially used a stent retriever for thrombectomy. Since an appropriately-sized stent for permanent treatment was not available, we innovatively maintained temporary revascularization with the stent retriever for 90 minutes. Here we demonstrate the adaptability of the stent retriever for emergency care. A 49-year-old man suddenly developed severe right hemiplegia and aphasia. Magnetic resonance imaging showed occlusion of a left internal carotid artery with moderate ischemic changes in the left hemisphere cortex. Angiography showed dissection of the left internal carotid artery at the cervical level and secondary thrombus formation extending into the left middle cerebral artery. We initially attempted thrombectomy with a stent retriever and achieved successful reperfusion in the middle cerebral artery. An appropriately-sized stent was not available in the hospital at that time. During the 90-minute wait, the stent retriever was kept in place and temporary angioplasty was performed in the internal carotid artery dissection to maintain blood flow. Eventually, the stent was delivered and permanent revascularization was achieved. While there is no standard treatment for arterial dissection, endovascular strategies like stenting have been demonstrated to be beneficial. The innovative use of stent retrievers for temporary angioplasty of dissected lesions underscores their efficacy in swift deployment and maintenance of uninterrupted blood flow, particularly during emergency thrombectomy.

## Introduction

Thrombectomies have become widely practiced emergency procedures to remove blood clots that cause embolisms. They can reveal the numerous causes of embolisms, including not only cardiogenic sources but also atherothrombotic arteriosclerosis and malignancy-associated Trousseau syndrome. Although internal carotid artery (ICA) dissection is rare, it accounts for up to 25% of stroke cases in young people [1]. In hospital settings, it is usually possible prepare a stent that fits the target vessel size immediately. However, thrombectomy is often performed in emergency situations when appropriate equipment may not be readily available. Stent retrievers, intended for thrombus extraction, can be used in these cases for temporary angioplasty for dissected lesions. They can be an effective and swift method that ensures uninterrupted blood flow.

## Case report

**Fig. 1:**
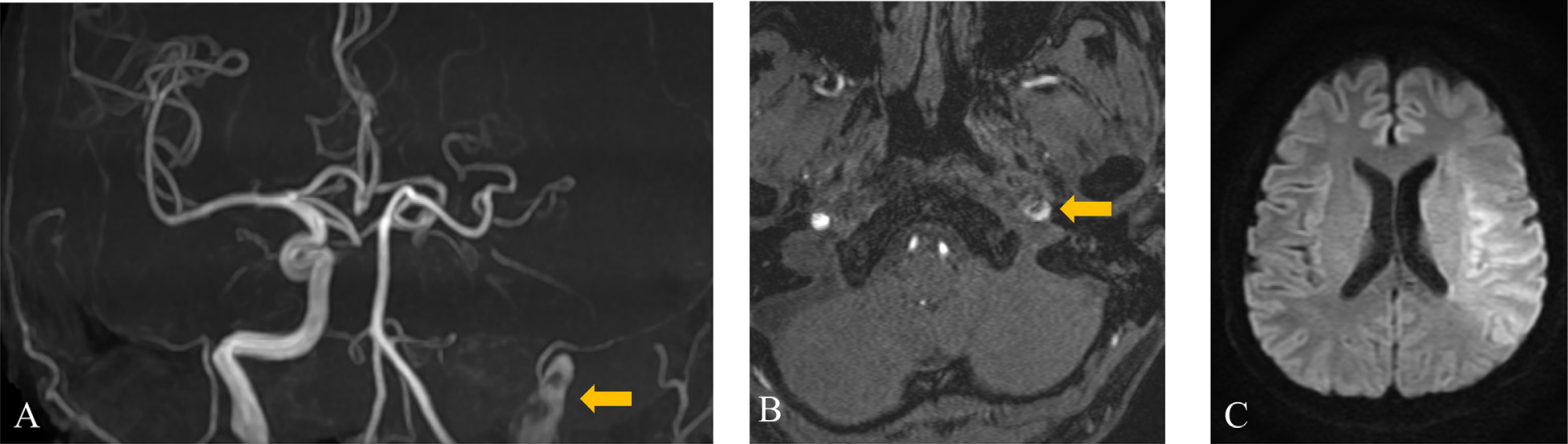
Preprocedure magnetic resonance imaging (MRI) of dissection of the internal carotid artery (ICA). (A and B) MRI revealed a subacute intramural hematoma in the wall of the right ICA (arrow). (C) MRI showed an acute ischemic lesion in the left cortex.

**Fig. 2:**
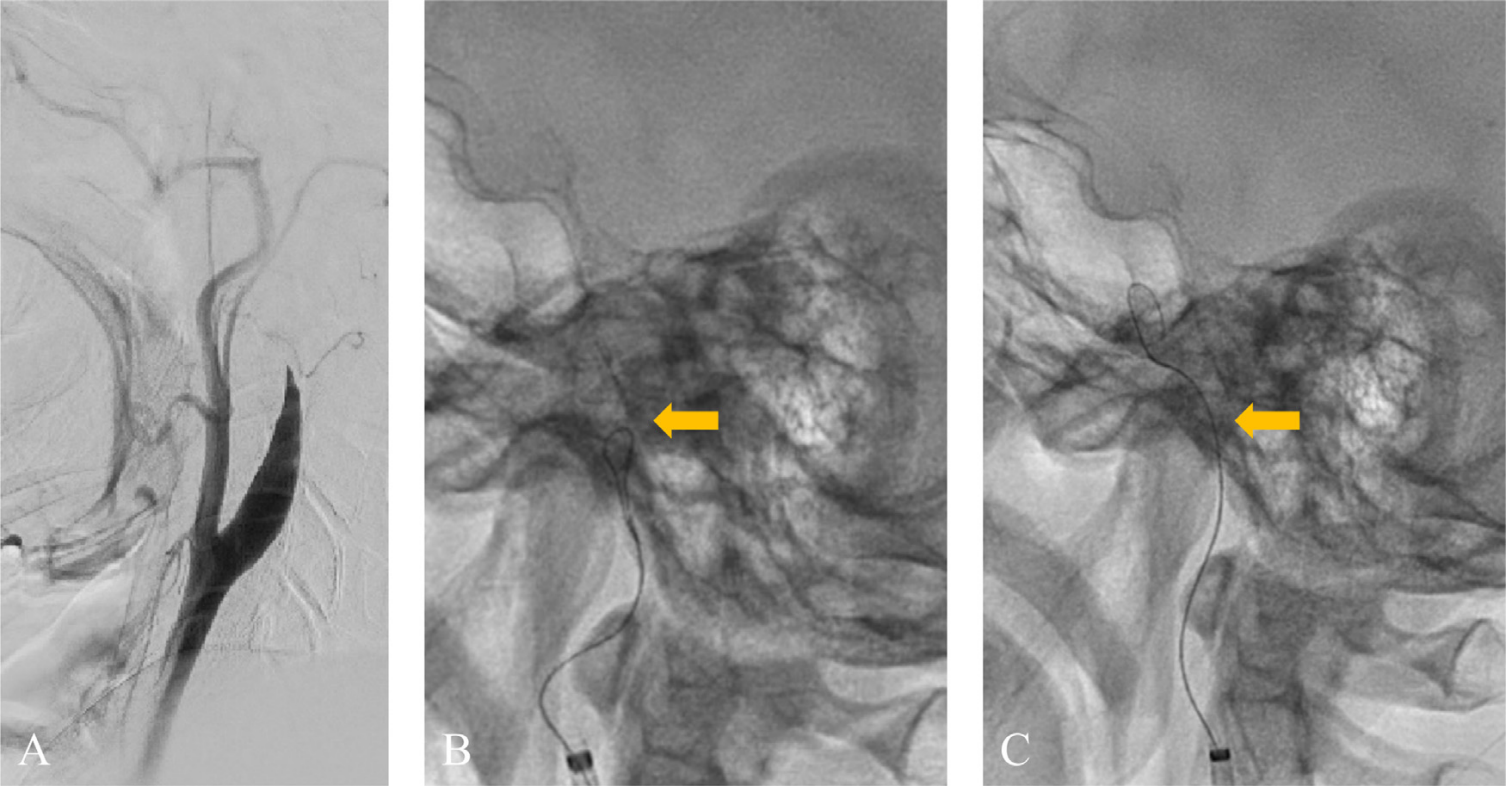
(A) A flame-like occlusion, typical of an acute dissection, is visible in the lateral view digital subtraction angiography (DSA) of the left carotid artery. (B) The microwire made several U-turns at the left ICA prepetrous portion before crossing (arrow). (C) After several attempts, the microwire crossed the prepetrous portion (arrow).

**Fig. 3:**
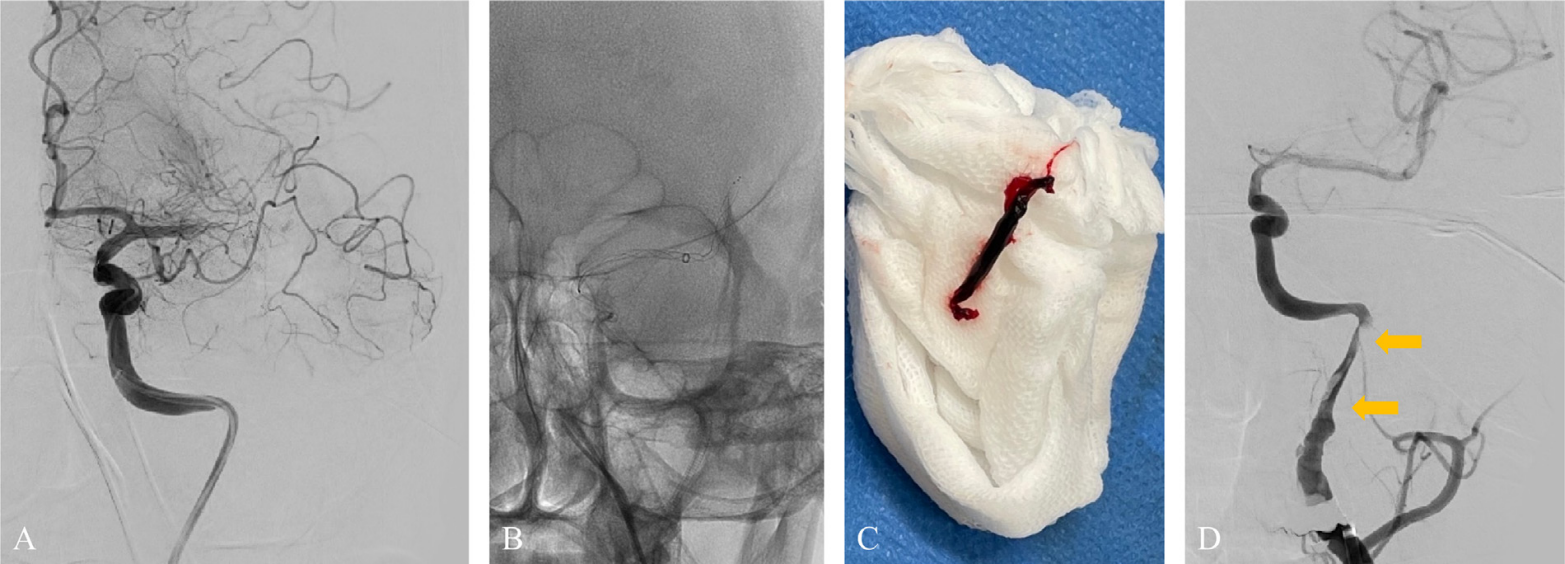
(A) Left ICA angiography confirmed MCA occlusion. (B) Thrombectomy was conducted. (C) A large clot was retrieved. (D) After thrombectomy, MCA occlusion was recanalized and the presence of the extracranial ICA dissection with a high-grade stenosis was confirmed (double arrow).

**Fig. 4:**
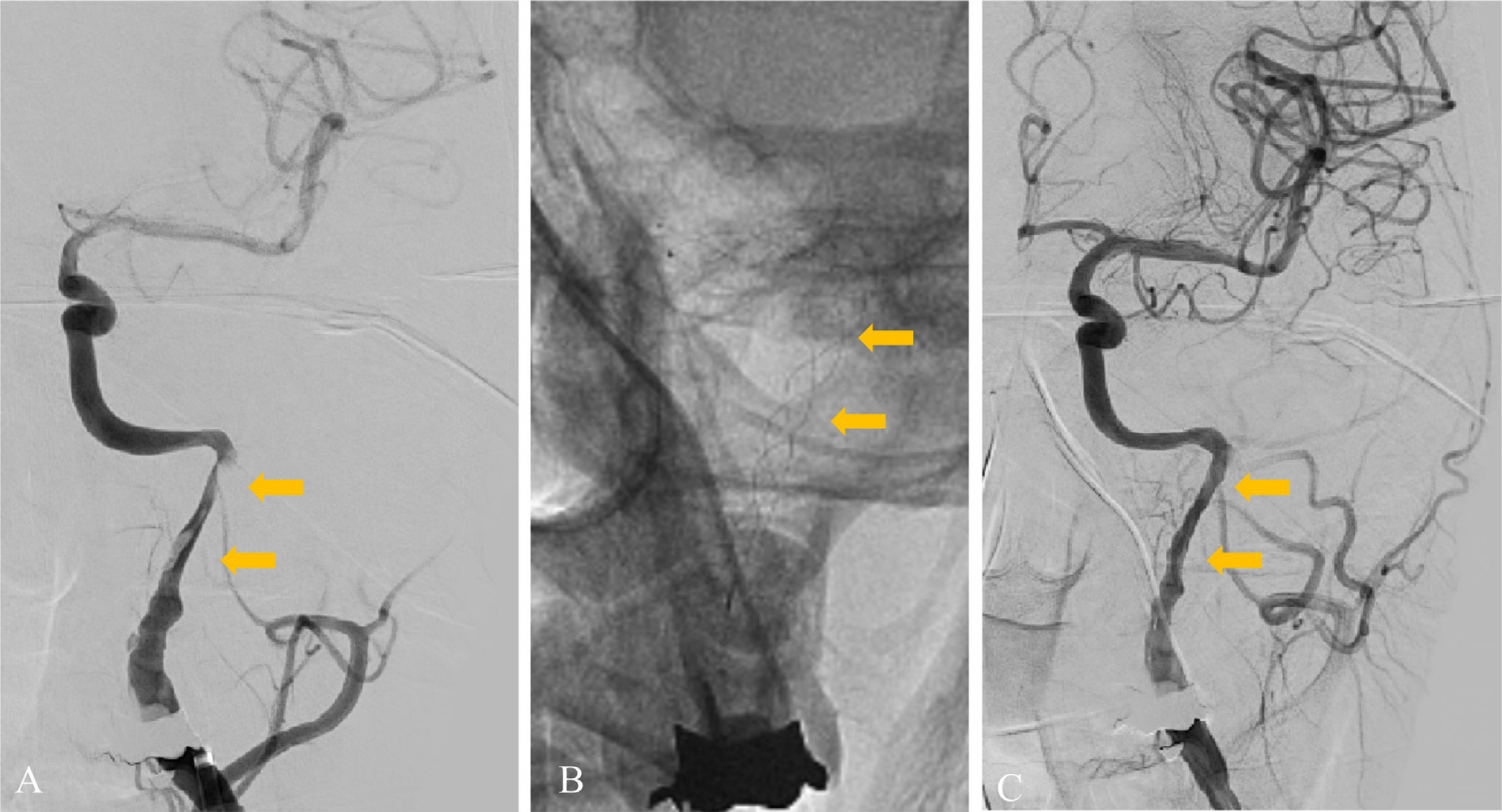
(A) A high-grade stenosis (double arrow) with poor peripheral perfusion is seen in the dissection of the left ICA. (B) A 4 × 41mm stent retriever was deployed at the dissection, including the high-grade stenosis segment (double arrow). (C) The stent retriever successfully restored clear flow (double arrow) and improved peripheral blood flow.

**Fig. 5:**
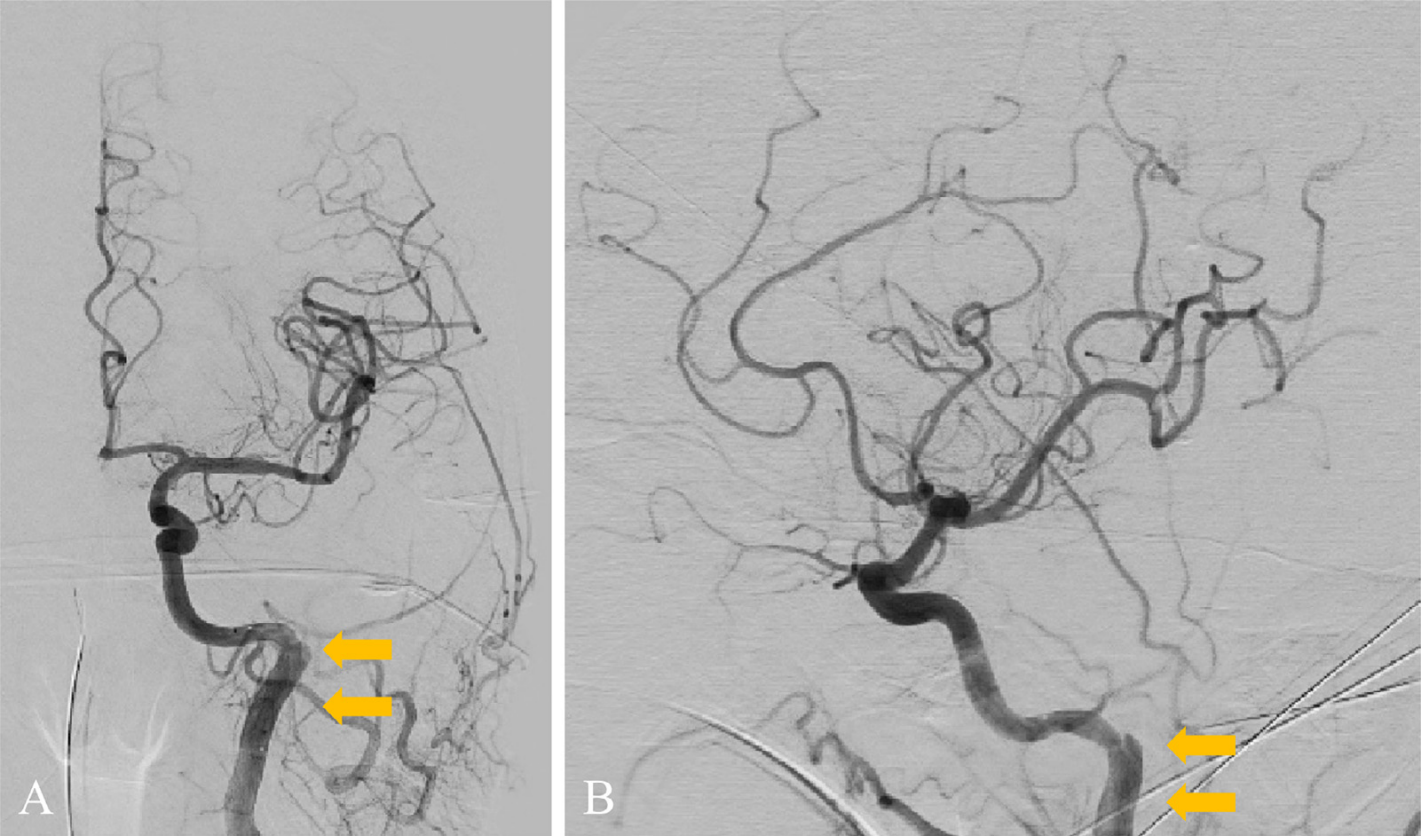
(A and B) The final angiography after stenting using a 6 × 30 mm stent and a 4.5 × 30 mm stent revealed sufficient reperfusion of the left ICA (double arrow).

A 49-year-old man with no medical or drug history worked for a transport company and returned to the office after completing his delivery duties. He suddenly collapsed in front of his colleagues. Upon arrival at our hospital, the patient exhibited severe right-hand hemiplegia and aphasia (National Institutes of Health Stroke Scale 22). Magnetic resonance imaging (MRI) revealed occlusion of the left ICA, intramural hematoma, and moderate infarction in the left insular cortex, centrum semiovale, and left parietal lobe (Fig. 1). During diagnostic angiography, we confirmed a tapered, flame-like occlusion at the cervical level, typical of an acute dissection of the left ICA (Fig. 2). We also observed a large vessel occlusion (LVO) (Fig. 2A). However, arterial dissection was not observed at that point. The first treatment goal was to remove the thrombus and open the arteries. A 9-French (Fr) introducer was placed in the right femoral artery. After systemic heparinization (intravenous injection at 5000 units, followed by continuous intravenous infusion of 1000 units/h to maintain an activated clotting time twice the patient's baseline level), a 9-Fr guiding catheter (Optimo FLEX, Tokai Medical Products, Aichi, Japan) was introduced into the left ICA. Initially, aspiration was conducted using balloon catheter dilatation, but the thrombus could not be removed. With a microcatheter (Trak21, Stryker, California, USA) and microwire (Synchro2 standard, Stryker, California, USA), the Synchro made several U-turns at the left ICA prepetrous portion before crossing (Figs. 2B and C). After several attempts to rotate the J-shaped wire, we guided the microcatheter to the M2 segment of the left middle cerebral artery (MCA). Thrombectomy was conducted using a stent retriever (4×41TrevoNXT, Stryker, California, USA) and an aspiration catheter (Vecta71, Stryker, California, USA). A significant number of soft, dark red thrombi were retrieved (Figs. 3A-C). Subsequent angiography revealed reperfusion of the left MCA. However, significant stenosis was noted in the more proximal left ICA prepetrous portion, along with peripheral blood flow stagnation (Fig. 3D). Based on vascular morphology, patient age, and the absence of other risk factors, the diagnosis was dissection. A secondary thrombus had formed at the dissection site that caused occlusion of the ICA (Fig. 4A). Therefore, angioplasty and stenting were imperative. Aspirin (200 mg) and prasugrel (20 mg) were administered via a gastric tube before stent implantation. However, an appropriately-sized stent for the diameter of the petrous portion of the vessel was not in stock, which caused a delay of approximately 90 min. This delay was due to the time it would take for a messenger to bring the appropriate device from another facility, prolonging cerebral hypoperfusion and potentially leading to an expansion of the cerebral infarction. This situation raised concerns about the availability of an appropriate device and the critical impact of such delays on patient outcomes. To maintain blood flow in the ICA during this time, the stent retriever used for thrombus retrieval was deployed again in the dissection. A stent was placed during the dissection to prevent reocclusion. To maintain blood flow in the ICA while waiting, a 300-cm microwire (CHIKAI) was passed parallel to the distal left M2 region of the MCA to secure the true lumen. Another catheter (Trak21MC + Synchro Standard) was then placed in the dissection with a 4 × 41 mm stent (Trevo) deployed (Fig. 4B). Although the stent was undersized for the vessel, anterior cerebral artery (ACA) was visualized after stenting. it successfully restored blood flow and improved peripheral blood flow (Fig. 4C). After the arrival of the stent, a precise 6 × 30-mm stent was placed. However, due to the inability of the shaft to surpass the curvature of the petrous portion, stenosis remained. The deployment of the flexible 4.5 × 30 mm-stent (Neuroform Atlas) from the stenosis to the periphery significantly improved vascular patency. Imaging of the left ICA confirmed improvement in cerebral blood flow. After a 30-min observation period, repeated imaging confirmed the absence of reocclusion, and the final left ICA angiography revealed complete reperfusion (Figs. 5A and B). No postoperative complications were observed, and no new lesions were identified on follow-up MRI. The patient was discharged with mild aphasia and was transferred to a rehabilitation hospital.

## Discussion

Arterial dissections account for 2% of strokes across all age groups, and they account for up to 25% of cases of stroke in individuals less than 45 years of age. In most cases, stroke is considered idiopathic and is more prevalent in younger individuals. Distal emboli may arise from clots formed in the low-flow zone of the false arterial lumen and the artery may eventually become occluded by a mural hematoma [[1], [2], [3], [4]]. The American Heart Association highlights embolism as the primary cause of ischemic symptoms in 85% of cases, which underscores the importance of antithrombotic therapy. However, thrombectomy may be appropriate in cases of cerebral LVO, and it is advisable to reestablish the intracranial vascular flow before treating the dissection [3,5]. No formal standards or guidelines for endovascular approaches or early antithrombotic treatment for ICA dissection haven been established [6,7]. The effectiveness of angioplasty and stenting at the dissection site is controversial in cases without poor peripheral perfusion; however, it is likely to be safe and beneficial in patients with reduced perfusion in the distal arteries and associated symptoms [1,3,[5], [6], [7], [8], [9], [10]]. In our case, after MCA thrombectomy was performed, the source of the embolism was found to be the ICA dissection. The dissection caused significant narrowing due to the compression of the true lumen by the false lumen at the dissection site, which resulted in reduced blood flow distal to the dissection. Revascularization with stent placement was urgently needed. However, because of the time constraints involved in procuring a stent that matched the blood vessel size, we innovatively utilized a stent retriever, typically employed for thrombectomy, as an alternate method. This decision highlights our adaptive approach to overcome an unexpected encounter with ICA dissection during thrombectomy. In a systematic review, the Time to Treat was relatively long, which extended the duration until reperfusion began. However, this was attributed to the potential delay caused by the time required to place a stent in the ICA. The discovery of ICA dissection in emergency situations and the need for an experienced endovascular therapist may have also contributed to the delay in the procedure [11]. The inability to prepare a stent that fits the vessel diameter immediately, as in this case, was another factor for delay. A systematic review of 141 patients from 31 studies who underwent endovascular management for extracranial dissection revealed a technical success rate of 99% and procedural complication rate of 1.3% [12]. In summary, the outcomes of stenting are highly positive; however, the principal challenge lies in the time required to reestablish blood flow in patients with cerebral perfusion insufficiency. A stent is a permanent device used to keep narrowed vessels open. Conversely, a stent retriever is a temporary device primarily used in thrombectomy to remove blood clots from occluded cerebral arteries, restoring blood flow in acute ischemic stroke patients. The fundamental difference lies in their application. Despite their different uses, stents and stent retrievers share a similar mesh-like design. This design enables stent retrievers, though primarily intended for clot removal, to temporarily expand the vessel during the retrieval process. This dual functionality underscores their role in acute ischemic stroke management, providing both clot removal and transient vessel dilation. The use of a stent retriever for revascularization is generally more flexible than that of a permanent stent and allows easier and quicker deployment, especially in flexed and tortuous vessels such as those in our case. Although the stent was slightly undersized, it was sufficient to diminish delayed blood flow and maintain antegrade flow. The clear visualization of the ACA following stenting indicates a rapid improvement in cerebral blood flow (CBF) [13]. It prevented further thrombus formation in the narrowed dissection area until antiplatelet drugs could be administered. However, this technique requires eventual replacement of the stent retriever with a permanent stent, which necessitates caution during the exchange. In our case, the true lumen of the dissection cavity could not be secured when extracting the stent retriever. The crossing of the dissected segment is the most important limiting factor for successful ICA recanalization [1,14]. One concern is that the subintimal space may be further enlarged when the true lumen is rewired and, in the worst case, the false lumen may further compress and obstruct the true lumen [2]. To ensure safety of the procedure, another wire was inserted parallel to the true lumen before deploying the stent retriever to ensure that the true lumen was always secured.

## Conclusion

Arterial dissection plays a critical role in stroke in younger patients. However, standardized treatments remain undefined. Endovascular strategies, notably stenting, prove beneficial, particularly during thrombectomy processes. The innovative use of stent retrievers, initially intended for thrombus extraction, as temporary angioplasty for dissected lesions underscores their efficacy in swift deployment and maintenance of uninterrupted blood flow.

## Patient consent

The authors certify that they have obtained all appropriate patient consent forms. In the form, the patient has given consent for their images and other clinical information to be reported in the journal. The patient understands that their name and initials will not be published and due efforts will be made to conceal their identity, but anonymity cannot be guaranteed.

## Submission statement

This manuscript is original and has not been submitted elsewhere in part or in whole.

## Previous presentations

None.
